# Unicystic Mural Ameloblastoma: An Unusual Case Report

**DOI:** 10.1155/2013/957418

**Published:** 2013-04-23

**Authors:** V. Nagalaxmi, Mithare Sangmesh, Kotya Naik Maloth, Srikanth Kodangal, Vani Chappidi, Stuti Goyal

**Affiliations:** Sri Sai College of Dental Surgery, H. No. 5-5-47/1, Mustafa Nagar, Khammam 507 001, Vikarabad, Andhra Pradesh, India

## Abstract

Ameloblastoma is a benign odontogenic neoplasm which frequently affects the mandible. The term ameloblastoma includes several clinicoradiological and histological types. Apart from the most commonly encountered clinicopathologic models, there are few variants, whose biological profile is unknown or not elicited. Among these types, unicystic ameloblastoma is the least encountered variant of the ameloblastoma. Unicystic ameloblastoma refers to those cystic lesions that show clinical, radiographic, or gross features of a jaw cyst but on histologic examination show a typical ameloblastomatous epithelium lining the cyst cavity, with or without luminal and/or mural tumor proliferation. Unicystic ameloblastoma is a less encountered variant of the ameloblastoma and is believed to be less aggressive. As this tumor shows considerable similarities with dentigerous cysts, both clinically and radiographically, the biologic behaviour of this tumor group was reviewed. Moreover, recurrence of unicystic ameloblastoma may be long delayed, and a long-term postoperative followup is essential for proper management of these patients. Here we are presenting a case of unicystic ameloblastoma in an 18-year-old female patient.

## 1. Introduction

Many benign lesions cause mandibular swellings, and these can be divided into odontogenic and nonodontogenic origin. The most common tumor of odontogenic origin is ameloblastoma which develops from epithelial cellular elements and dental tissues in their various phases of development. More than 80% of all ameloblastomas are solid or multicystic variants, with unicystic ameloblastoma being an important clinicopathologic form of ameloblastoma and occupying the remaining 20% of the cases along with peripheral ameloblastoma [1]. Unicystic ameloblastoma, a variant of ameloblastoma, was first described by Ackermann et al. in 1988 [2]. Unicystic ameloblastoma (UCA) is the most common term used to designate its different pathological entities. Sometimes these can present as a multilocular radiolucency which makes the use of the term “cystic ameloblastoma” more appropriate. However, some authors still believe that the notion that cystic ameloblastomas can have a “true” clinically multicystic pattern is arguable and contend with the use of the term “unicystic ameloblastoma” [3, 4].

The unicystic ameloblastoma is a less encountered variant of the ameloblastoma, referring to those cystic lesions that show clinical and radiographic characteristics of an odontogenic cyst but in histologic examination show a typical ameloblastomatous epithelium lining part of the cyst cavity, with or without luminal and/or mural tumor proliferation [5]. This paper illustrates a case of unicystic (mural) ameloblastoma of the mandible in an 18-year-old female.

## 2. Case Report

**Figure 1:**
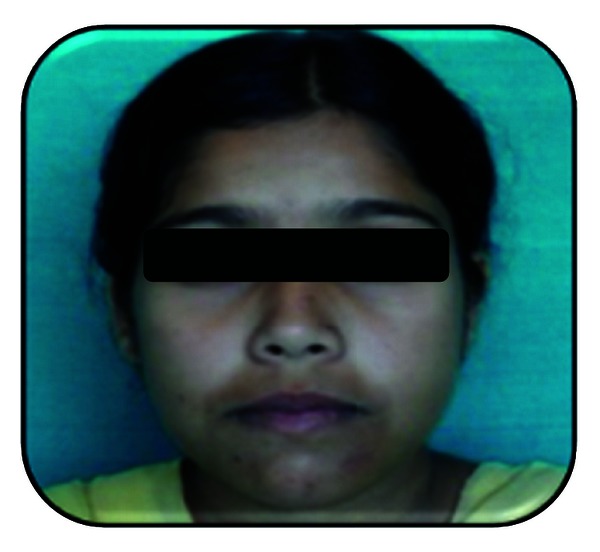
Preoperative extraoral photograph.

**Figure 2:**
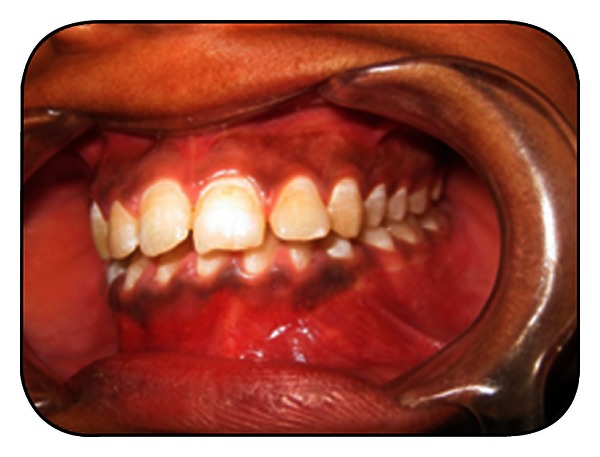
Preoperative intraoral photograph.

An 18-year-old female patient reported to our outpatient department with a chief complaint of swelling in the lower left front teeth region, for 3 months. Patient was apparently well 3 months back and noticed a swelling and displacement of teeth in the left lower front tooth region and reports of having pain in the same region, for 3 months. Pain was of dull aching type, which was intermitted, and it aggravates on putting mastication and relieves on rest. Pain was not associated with fever and no medication was taken. On extraoral examination a diffuse swelling was seen on the lower third of the face extending onto the left side with mild obliteration of mentolabial sulcus which is measuring about app 4 × 3 cm in size. Overlying skin was normal; no visible pulsations and no discharge were seen. On palpation, the swelling was firm in consistency and tender; no local rise of temperature was felt. It was nonpulsatile and noncompressible, and no discharge was present. A single left submandibular lymph node of size measuring about 0.5 × 0.5 cms in size was palpable, which was firm in consistency, mobile, and nontender (Figure 1). On intraoral examination, a single diffuse swelling was seen in the mandibular left buccal vestibule irt 31, 32, 33, and 34 regions measuring approximately 4 × 2 cms. It extends anterioposteriorly—from the mandibular labial frenum to 35 and superior-inferiorly from the attached gingival to the labial vestibule. Expansion of the lingual cortical plate was seen irt to 31, 32, and 33 (33 is missing). Neither discharge nor pulsations were seen. On palpation, crepitus was felt on the lingual cortical plate. Tenderness was elicited on palpation irt 33, 34, and 35. The swelling was firm in consistency, and surface was smooth. It was nonfluctuant and nonreducible and no discharge was noticed, non were pulsations felt (Figure 2). On needle aspiration, brown yellow fluid was aspirated. Based on the patient's history and clinical finding, the diagnosis was given as dentigerous cyst.

## 3. Differential Diagnosis

In differential diagnosis, ameloblastoma, calcifying epithelial odontogenic tumor (CEOT), odontogenic keratocyst (OKC), central giant cell granuloma (CGCG), odontogenic myxoma were considered but features like old age, site, multilocularity of ameloblastoma, and impacted lower canine made us deviate from ameloblastoma. In CEOT focal areas of calcifications are seen, but in our case we see unilocular radiolucency without any radio opaque flecks. Linear expansion of OKC through medullary spaces without any buccolingual expansion ruled out OKC. Lesions like CGCG and odontogenic myxoma were also ruled out based on their clinical and radiological features [6–8]. 

## 4. Investigations

**Figure 3:**
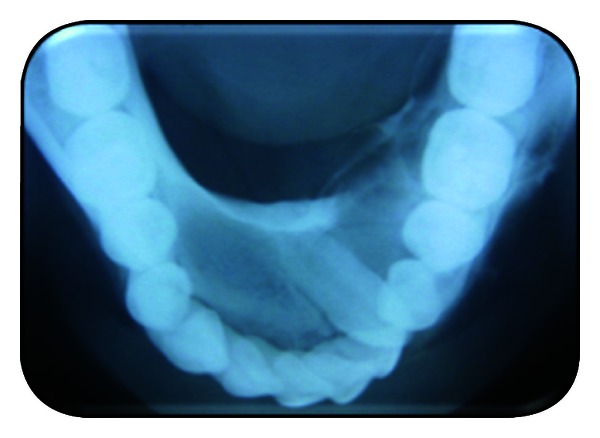
Occlusal radiograph.

**Figure 4:**
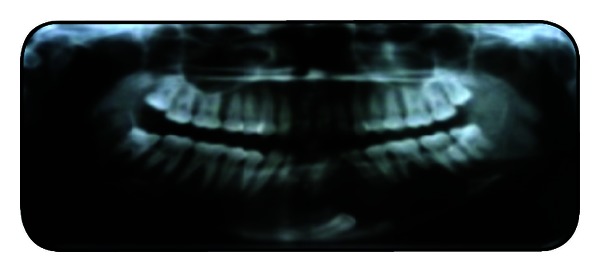
OPG radiograph.

**Figure 5:**
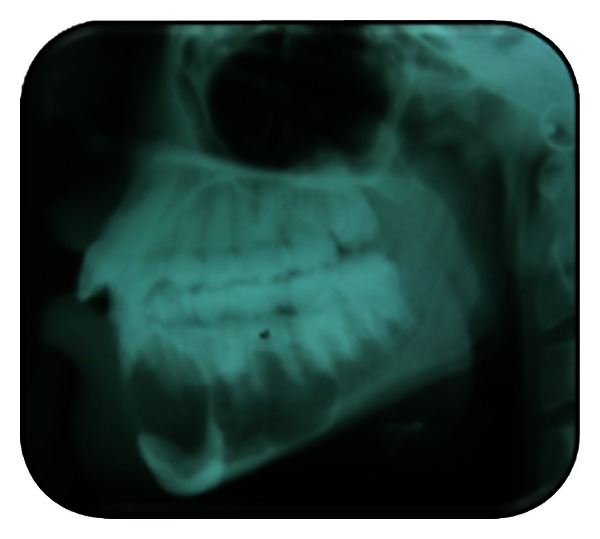
Lateral cephalogram radiograph.

**Figure 6:**
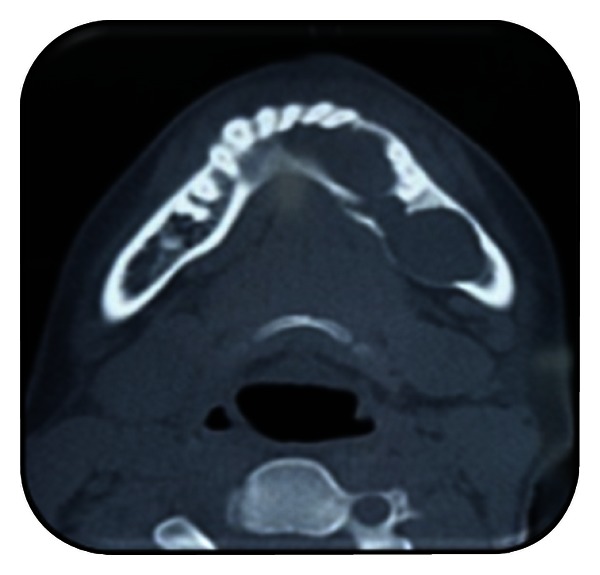
CT scan (axial view).

**Figure 7:**
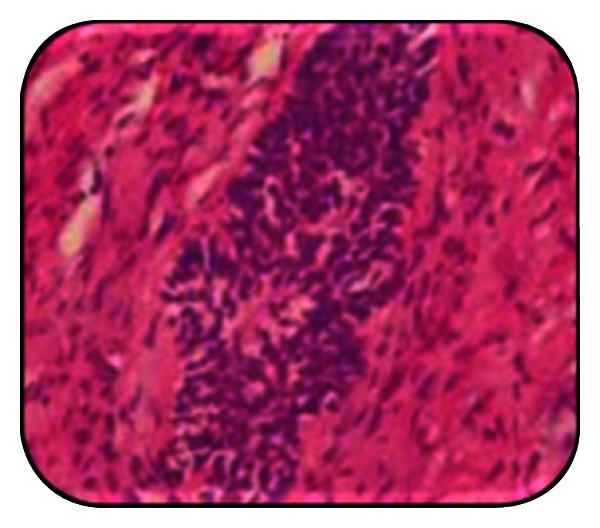
Histopathology.

Routine investigations like complete blood picture were done, which were normal. On FNAC, a brown yellow fluid was aspirated. Radiographs were taken. Mandibular occlusal view reveals impacted 33 and expansion of lingual cortical plates irt 32, 33, 34, 35, and 36 and mild expansion of buccal cortical plate (Figure 3). OPG reveals a well-defined multilocular radiolucency with internal bony septae formation and impacted 33. Displacements of teeth irt 35, 34, 32, 31, 41, 42, and 43 were seen, with root resorption of 36 (Figure 4). A lateral cephalogram reveals a clear multilocular radiolucency with septae formation (Figure 5). A CT scan reveals expansion and perforation of the cortical plates and the extent of lesion (Figure 6). Incisional biopsy was done and submitted for histopathological examination. The biopsy tissue shows multiple cystic lesions and collagenous wall of varying thickness. The cyst is lined by odontogenic squamous epithelium of varying thickness with nuclear palisading along the margins and loose stellate reticulum. They contain moderate to abundant pale acidophilic vacuolated cytoplasm and round to oval vesicular nucleus. There are thin plates of lamellar bone with focal osteoid deposition and patchy areas of hemorrhage. There are focal epithelial invaginations associated with desmoplastic fibrosis (Figure 7). Based upon the radiological and histopathological report, the case was diagnosed as mural ameloblastoma.

## 5. Treatment 

**Figure 8:**
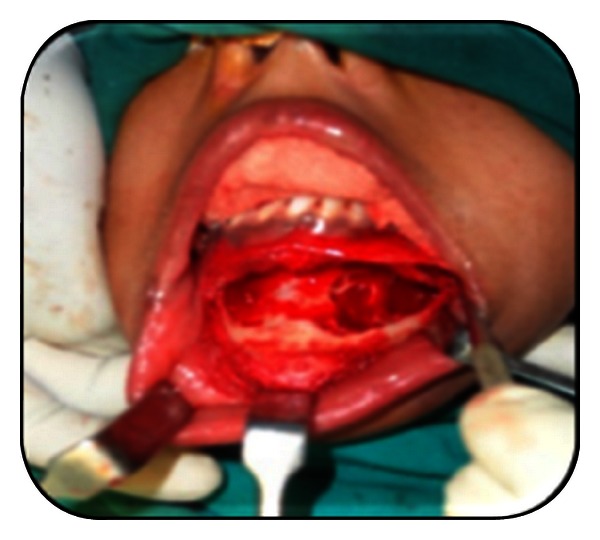
Intraoperative enucleation.

**Figure 9:**
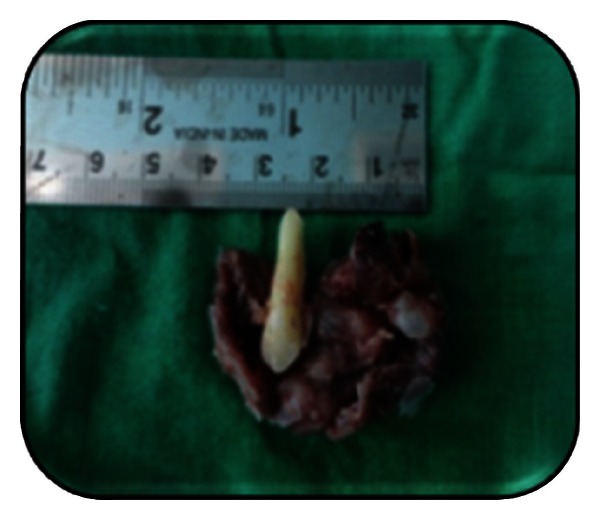
Intraoperative enucleation.

The lesion was treated conservatively with careful *enucleation* (Figures 8 and 9). 

## 6. Discussion

Ameloblastoma is a benign, locally aggressive odontogenic neoplasm with variable clinical expression and accounts for 1% of all cysts/tumors of jaws and 18% of all odontogenic neoplasms. It is typically slow growing, locally aggressive and rarely metastasizes but has a high rate of recurrence (55–90%) if not removed adequately.

As per the WHO system of 2003, ameloblastoma is classified based on differences in biologic behavior, treatment plan and recurrence rate as follows:

1. classic solid/multicystic ameloblastoma,
2. unicystic ameloblastoma,
3. peripheral ameloblastoma,
4. desmoplastic ameloblastoma, including the so-called hybrid lesions [3].

Unicystic ameloblastoma (UCA) is a rare type of ameloblastoma, accounting for about 6% of ameloblastomas. It usually occurs in a younger age group of 16–20 years, with about 50% of the cases occurring in the second decade of life as in our case [9, 10]. The gender distribution shows a slight male predilection with a male to female ratio of 1.6 : 1. However, when the tumor is not associated with an unerupted tooth, the gender ratio is reversed to a male to female ratio of 1 : 1.8 [11]. More than 90% are located in the mandible in the posterior region, followed by the parasymphysis region, the anterior maxilla, and the posterior maxilla [9]. UCA is usually asymptomatic, although a large tumor may cause painless swelling of the jaws with facial asymmetry [9]. Mucosal ulceration is rare but may be caused by continued growth of the tumor [12]. The clinical and radiographic findings in most cases of unicystic ameloblastoma suggest that the lesion is an odontogenic cyst, particularly dentigerous cyst. However, few are not associated with impacted teeth which are called nondentigerous variant [12]. The mean age of nonimpacted tooth-related cystic ameloblastoma was 5 years in comparison to 16.5 years for the impacted tooth-related variant [2]. Most of the UCAs are associated with an impacted tooth, the mandibular third molar being involved most often. But in our case it was associated with impacted mandibular canine, and it is a dentigerous variant. These findings correlate with those reported by Philipsen et al. and Ackermann et al.

The radiographic appearance of UCAs has been divided into 2 main patterns: unilocular and multilocular, and these have clear preponderance for the unilocular pattern. This preponderance is predominantly marked for the dentigerous variant, where the unilocular to multilocular ratio is 4.3 : 1, and for the nondentigerous type, this ratio is 1.1 : 1 [4, 13]. The involved teeth show varying degrees of root resorption [3].

Eversole et al. and Paikkatt et al. identified predominant radiographical patterns for UCA: unilocular, scalloped macromultilocular, pericoronal, interradicular, or periapical expansile radiolucencies [4, 14]. Our case study had a peculiar radiographic presentation of multilocular radiolucency crossing the midline of the mandible. The early ameloblastic changes within the cyst wall were first described by Vickers and Gorlin in 1970, and their histologic criteria for the diagnosis of unicystic ameloblastoma includes a cyst lined by ameloblastic epithelium with a tall columnar basal layer, subnuclear vacuoles, reverse polarity of hyperchromatic nucleus, and a thin layer of oedematous, degenerating stellate reticulum-like cells on the surface [15]. The mural extension into the cystic wall is the frequently seen feature, and the term mural UCA is used when the thickened lining (either plexiform or follicular) penetrates the adjacent capsular tissue [1, 9].

In a clinicopathologic study of 57 cases of unicystic ameloblastoma, Ackermann classified this entity into the following three histologic groups [2, 16]:

 Group I—luminal UA (tumor confined to the luminal surface of the cyst);
 Group II—intraluminal/plexiform UA (nodular proliferation into the lumen without infiltration of tumor cells into the connective tissue wall);
 Group III—mural UA (invasive islands of ameloblastomatous epithelium in the connective tissue wall not involving the entire epithelium). According to this classification, our case study belongs to Group III.

Histologic subgrouping by Philipsen and Reichart has also been described:

 Subgroup 1—luminal UA;
 Subgroup 1.2—luminal and intraluminal;
 Subgroup 1.2.3—luminal, intraluminal and intramural;
 Subgroup 1.3—luminal and intramural.

A definitive diagnosis of unicystic ameloblastoma can only be done by histological examination of the entire lesion and cannot be predicted preoperatively on clinical or radiographic grounds. As preoperative incisional biopsy is not representative of the entire lesion, it may result in an incorrect classification. The epithelial lining of a UCA is not always uniformly characteristic and is often lined partly by a nonspecific thin epithelium that mimics the dentigerous cyst lining. Thus, true nature of the lesion becomes evident only after enucleation when the entire specimen is available for microscopy [12]. The pathogenesis of cystic ameloblastomas remains obscure. Whether UCA originates *de novo* as a neoplasm or whether it is a result of neoplastic transformation of nonneoplastic cyst epithelium has long been debated. Some investigators believe that UCA arises from preexisting odontogenic cysts, in particular a dentigerous cyst, while others maintain that it arises *de novo.* Ackermann et al. (1988) and Robinson and Martinez (1977) argued that as the epithelium of odontogenic cysts and ameloblastomas have a common ancestry, a transition from a nonneoplastic to a neoplastic one could be possible, even though it occurs infrequently [2, 17].

Leider et al. (1985) proposed three pathogenic mechanisms for the evolution of UA [18].

1. The reduced enamel epithelium which is associated with a developing tooth undergoes ameloblastic transformation with subsequent cystic development.
2. Ameloblastomas arise in dentigerous cysts or in others in which the neoplastic ameloblastic epithelium is preceded temporarily by a nonneoplastic stratified squamous epithelial lining.
3. A solid ameloblastoma undergoes cystic degeneration of the ameloblastic islands, with subsequent fusion of multiple microcysts and develops into unicystic lesions.

Several attempts have been made in the past to distinguish the lining of the UCAs from that of odontogenic cysts. However, immunohistochemical markers like lectins (*Ulex europaeus* agglutinin I and *Bandeiraea simplicifolia* agglutinin I) and proliferating cells (proliferating cell nuclear antigen (PCNA) and Ki-67) may assist in their differential diagnosis [19]. However, Eversole et al. contend that currently unaided histologic assessment for UCA remains the gold standard for diagnosis, because of a variable response of UCA to tissue markers. Histologically, the minimum criteria for diagnosing a lesion as UCA are the demonstration of a single cystic sac lined by odontogenic (ameloblastomatous) epithelium often seen only in focal areas [20]. Treatment planning depends on the histological type of UA. The UA which is diagnosed as subgroups 1 and 1.2 may be treated conservatively (careful enucleation), whereas Subgroups 1.2.3 and 1.3 should be treated aggressively [11]. The histological typing of the current case was 1.2 and hence, the lesion was treated conservatively with careful enucleation. The recurrence rate for UAs after conservative surgical treatment (curettage or enucleation) is generally reported to be 10–20% [11] and on average, less than 25% [10]. This is considerably less than 50–90% recurrence rates which are noted after the curettage of conventional solid or multicystic ameloblastomas [11, 21]. Lau and Samman reported recurrence rates of 3.6% for resection, 30.5% for enucleation alone, 16% for enucleation followed by Carnoy's solution application, and 18% by marsupialisation followed by enucleation (where the lesion is reduced in size) [22].

**Figure 10:**
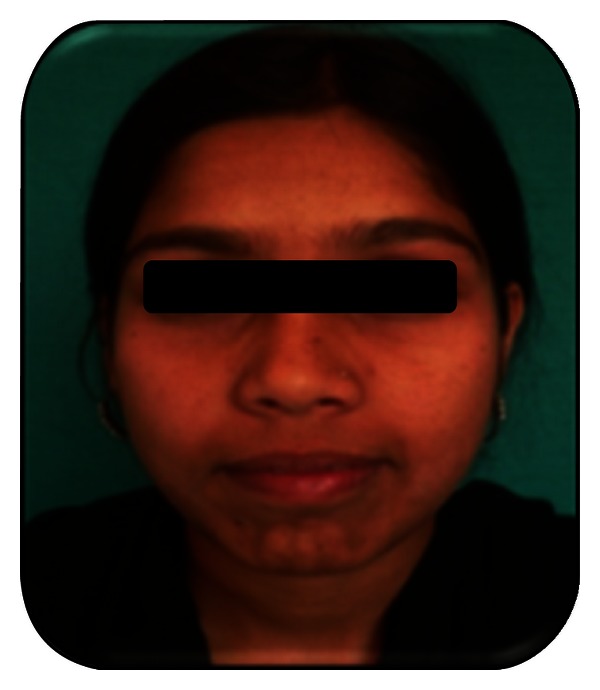
Postoperative photograph.

**Figure 11:**
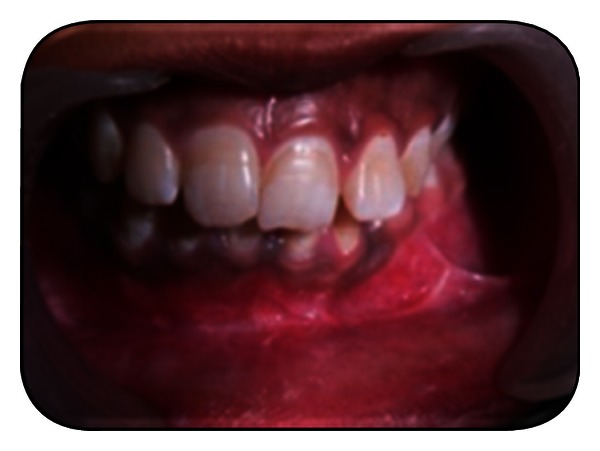
Postoperative photograph.

Whatever surgical approach the surgeon decides to take, long-term followup is mandatory as recurrence of unicystic ameloblastoma may be long delayed. The case was followed for 9 months; there was no recurrence noted till now (Figures 10 and 11). 

## 7. Conclusion

The diagnosis of unicystic ameloblastoma was based on clinical, radiological, histopathologic, and CT features. It is a tumor with a strong propensity of recurrence, especially when the ameloblastic focus penetrates the adjacent tissue from the wall of the cyst. Radiographically, most of ameloblastomas show multilocularity, whereas unicystic ameloblastomas show a single large unilocular radiolucency. Very rarely, we come across a case with presentation of both multilocular and unicystic type in the same person crossing midline. Unicystic variant of ameloblastoma with aggressive histologic behaviour also might be successfully treated with marsupialisation with subsequent enucleation, and this approach can be considered as an alternative to resection.
